# Whole-Exome Sequencing Identifies Novel Compound Heterozygous *ZNF469* Mutations in Two Siblings with Mild Brittle Cornea Syndrome

**DOI:** 10.1007/s00223-020-00721-3

**Published:** 2020-07-15

**Authors:** Tim Rolvien, Uwe Kornak, Stephan J. Linke, Michael Amling, Ralf Oheim

**Affiliations:** 1 Department of Osteology and Biomechanics, University Medical Center Hamburg-Eppendorf, Lottestr. 59, 22529 Hamburg, Germany; 2 Department of Orthopedics, University Medical Center Hamburg-Eppendorf, Hamburg, Germany; 3 National Bone Board, Martin Zeitz Center for Rare Diseases, University Medical Center Hamburg-Eppendorf, Hamburg, Germany; 4 Institute of Medical Genetics and Human Genetics, Charité-Universitätsmedizin Berlin, Berlin, Germany; 5 Berlin-Brandenburg School for Regenerative Therapies, Charité-Universitätsmedizin Berlin, Berlin, Germany; 6 FG Development and Disease, Max Planck Institute for Molecular Genetics, Berlin, Germany; 7 Department of Ophthalmology, University Medical Center Hamburg-Eppendorf, Hamburg, Germany

**Keywords:** Brittle cornea syndrome, ZNF469, Whole-exome sequencing, Osteogenesis imperfecta, Ehlers-Danlos syndrome

## Abstract

Connective tissue diseases, including osteogenesis imperfecta (OI) and Ehlers-Danlos syndrome (EDS), exhibit a high degree of clinical and genetic heterogeneity. We report two sisters with blue sclerae, joint hypermobility and hearing loss. Whole-exome sequencing identified two compound heterozygous *ZNF469* loss-of-function mutations due to a frameshift. Since these findings indicate the presence of brittle cornea syndrome (BCS), we performed ocular optical coherence tomography (OCT) and pachymetry, which revealed a moderate decrease in corneal thickness. While only one traumatic fracture was observed in each of the patients, a detailed skeletal assessment indicated no specific patterns of bone mass and microstructure reduction as well as normal bone turnover markers. Taken together, our findings point to a mild form of brittle cornea syndrome with a phenotype compatible with the extraskeletal features of OI but also with EDS.

**Electronic supplementary material:**

The online version of this article (10.1007/s00223-020-00721-3) contains supplementary material, which is available to authorized users.

## Introduction

Connective tissue diseases, including osteogenesis imperfecta (OI) and Ehlers-Danlos syndrome (EDS), exhibit not only a high degree of clinical but also genetic heterogeneity. OI is a well-described genetic skeletal disorder defined by increased bone fragility, which is most commonly caused by heterozygous mutations in two genes encoding type 1 collagen (*COL1A1*/*COL1A2*), however, many other forms and gene mutations have been identified [1]. Besides bone fragility, a number of extraskeletal symptoms including blue sclerae, joint hypermobility and hearing loss have been described [1]. Joint hypermobility is primarily a typical sign of EDS, a genetic connective tissue disorder caused by specific collagen mutations (type I, III or V) or other mutations in genes involved in collagen production or processing [2, 3]. While many atypical forms (both clinically and genetically) of OI and EDS have been reported, molecular analyses may also result in the detection of other connective tissue diseases. Zinc-finger proteins (ZNFs) are involved in a variety of cellular processes that occur via different molecular mechanisms [4]. Mutations in *ZNF469* cause brittle cornea syndrome (BCS) [5, 6], a multisystem connective tissue disorder primarily associated with corneal thinning but also blue sclerae and joint hypermobility [7]. BCS may also be caused by mutations in the gene encoding the transcription factor *PRDM5*. Although the exact molecular mechanisms for BCS pathogenesis remain unknown, *ZNF469* and *PRDM5* have been found to result in reduced expression of the same extracellular matrix (ECM) genes (e.g., *COL11A1*) [8]. In this report, we present the clinical and molecular features of two sisters with suspect of an inherited connective tissue diseases, who were later correctly diagnosed with a mild form BCS by extended diagnostics including whole exome sequencing.

## Patients and Methods

Here, we report two sisters (II.1, 47 years and II.2, 44 years) with blue sclerae, joint hypermobility and hearing impairment since childhood. Due to these specific clinical manifestations, which occurred at an early onset, the suspicion of a hereditary disorder was raised. Initially, OI had been suspected due to the presence of extraskeletal OI features, but had not been genetically confirmed. However, the sisters had suffered from only one traumatic fracture each (II.1 forearm, age 13; II.2 tibia, age 42). The further clinical history was uneventful. Both sisters were premenopausal, had a balanced diet, received no medication (especially no medication that could affect bone turnover or hearing) and were both normally physically active.

After exclusion of known disease genes for osteogenesis imperfecta and other skeletal and connective tissue diseases in the index case using a custom designed gene panel (Sure Select, Agilent) including 386 genes, whole-exome sequencing was carried out in the index patient and the unaffected parents (Human all exon V6, Agilent). Sequencing was performed on a HiSeq 2500 sequencing machine (Illumina, San Diego, CA, USA). Data were analyzed by the software tools GeneTalk [9] and MutationDistiller [10]. The variants were prioritized using the phenotype terms blue sclerae (HP:0000592), hypermobility of joints (HP:0001382), and hearing impairment (HP:0000365). *ZNF469* variants were ranked among the top 10 variants. The pathogenicity of the prioritized variants was judged using MutationTaster [11].

The Beighton Score was used to assess joint hypermobility [12]. To further investigate the ear and cornea phenotype, audiometry as well as ocular optical coherence tomography (OCT) and Pentacam corneal pachymetry were performed. In order to further analyze bone status, areal bone mineral density (aBMD) was evaluated in the lumbar spine and the left hip using dual energy X-ray absorptiometry (DXA, Lunar iDXA, GE Healthcare; Madison, WI, USA). Bone microstructure was analyzed in the non-dominant (left) distal radius and the contralateral distal tibia using high-resolution peripheral quantitative computed tomography (HR-pQCT; XtremeCT, Scanco Medical, Switzerland). Serum and urinary biochemical bone turnover markers including calcium, 25-hydroxyvitamin D, parathyroid hormone (PTH), osteocalcin, bone-specific alkaline phosphatase (BAP) and deoxypyridinoline (DPD) were assessed.

## Results

**Fig. 1:**
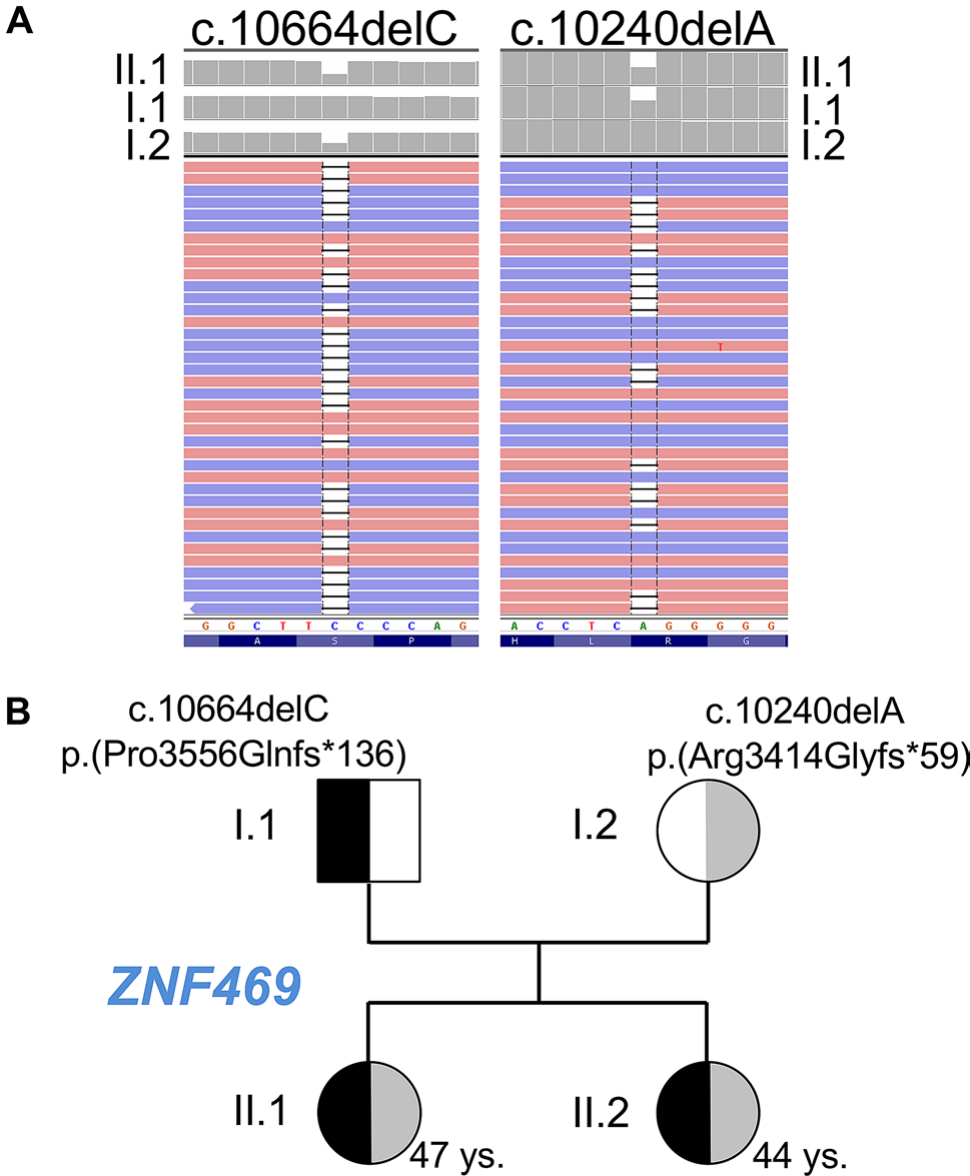
Compound heterozygous *ZNF469* mutations of two patients with mild brittle cornea syndrome. **a** Integrated Genome Viewer presentation demonstrating the compound heterozygosity of the *ZNF 469* mutations c.10664delC p.(Pro3556Glnfs*136) and c.10240delA p.(Arg3414Glyfs*59). **b** Pedigree

After all known OI genes were excluded by gene panel analysis, whole-exome sequencing of the index patient (II.1) and both parents revealed two heterozygous variants c.10664delC p.(Pro3556Glnfs*136) and c.10240delA p.(Arg3414Glyfs*59) in the *ZNF469* gene (Fig. 1a, b). Each of the variants was found heterozygous in one parent, and both variants were also detected in the sister (II.2) (Fig. 1a, b). The variants, which were confirmed by Sanger sequencing in all four family members, have not been reported before, but the frameshift effect of the single nucleotide deletions make a loss-of-function very likely. Both parents did not show any phenotypic abnormalities supporting the recessive inheritance.

The variants were ranked class V (pathogenic) according to ACMG criteria (PVS1, PM2, PM3, PP1, PP3) [13].

The two affected sisters presented with a normal height (II.1: 157 cm, 10. to 25. percentile; II.2: 165 cm, 50. to 75. percentile), blue sclerae and notable joint hypermobility. In fact, the Beighton score was 9/9 (II.1) and 8/9 (II.2) indicating severe hypermobility including the ability to bend the knees and elbows backwards (hyperextension in both sisters, knee: 20°, elbow: 30°). Furthermore, both sisters were affected by mild hip dysplasia (Crowe type I), which was currently asymptomatic. Patient II.2 also suffered from recurrent patella luxation, which was treated surgically by reconstruction of the medial patellofemoral ligament.

**Fig. 2:**
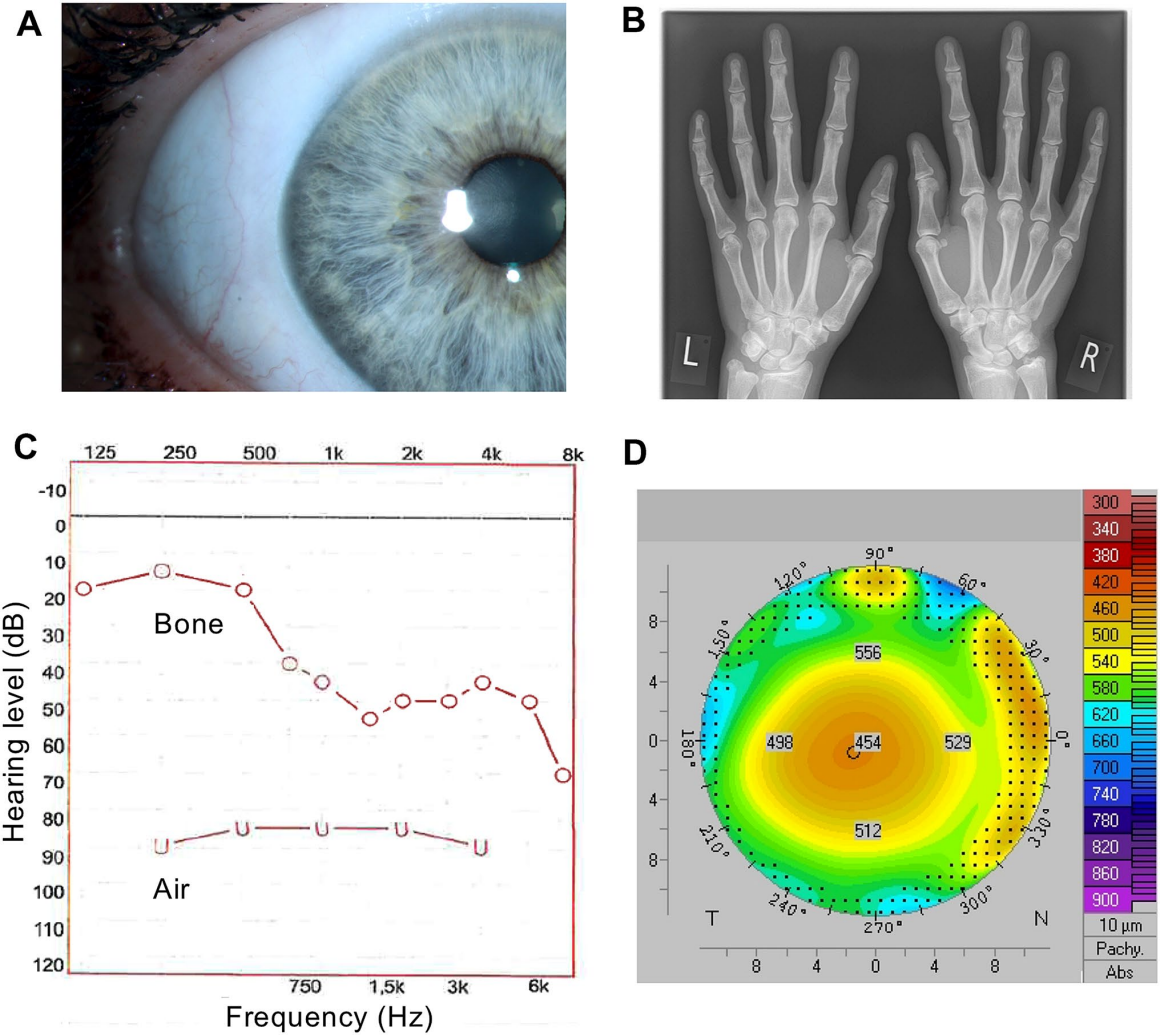
Clinical phenotype. **a** Blue sclera exemplary shown for patient II.1 (Slit lamp photograph). **b** Hand radiograph demonstrating long fingers. **c** Audiometry showing a mixed sensorineural and conductive hearing loss. **d** Corneal pachymetry showing moderately reduced corneal thickness

After detection of the *ZNF469* variants, the patients were closely reexamined for other typical features of brittle cornea syndrome (BCS). The blue sclerae were confirmed in both patients by several expert clinicians and by using a split lamp (Fig. 2a). Radiographs of the hands indicated rather long fingers without deformities (Fig. 2b). A combined sensorineural and conductive hearing loss was confirmed by audiometry (Fig. 2c). Ocular optical coherence tomography (OCT) and Pentacam pachymetry/tomography revealed a reduction in corneal thickness of 453 µm (II.1) and 456 µm (II.2) without the suspicion of keratoconus or keratoglobus (Fig. 2d).

**Fig. 3:**
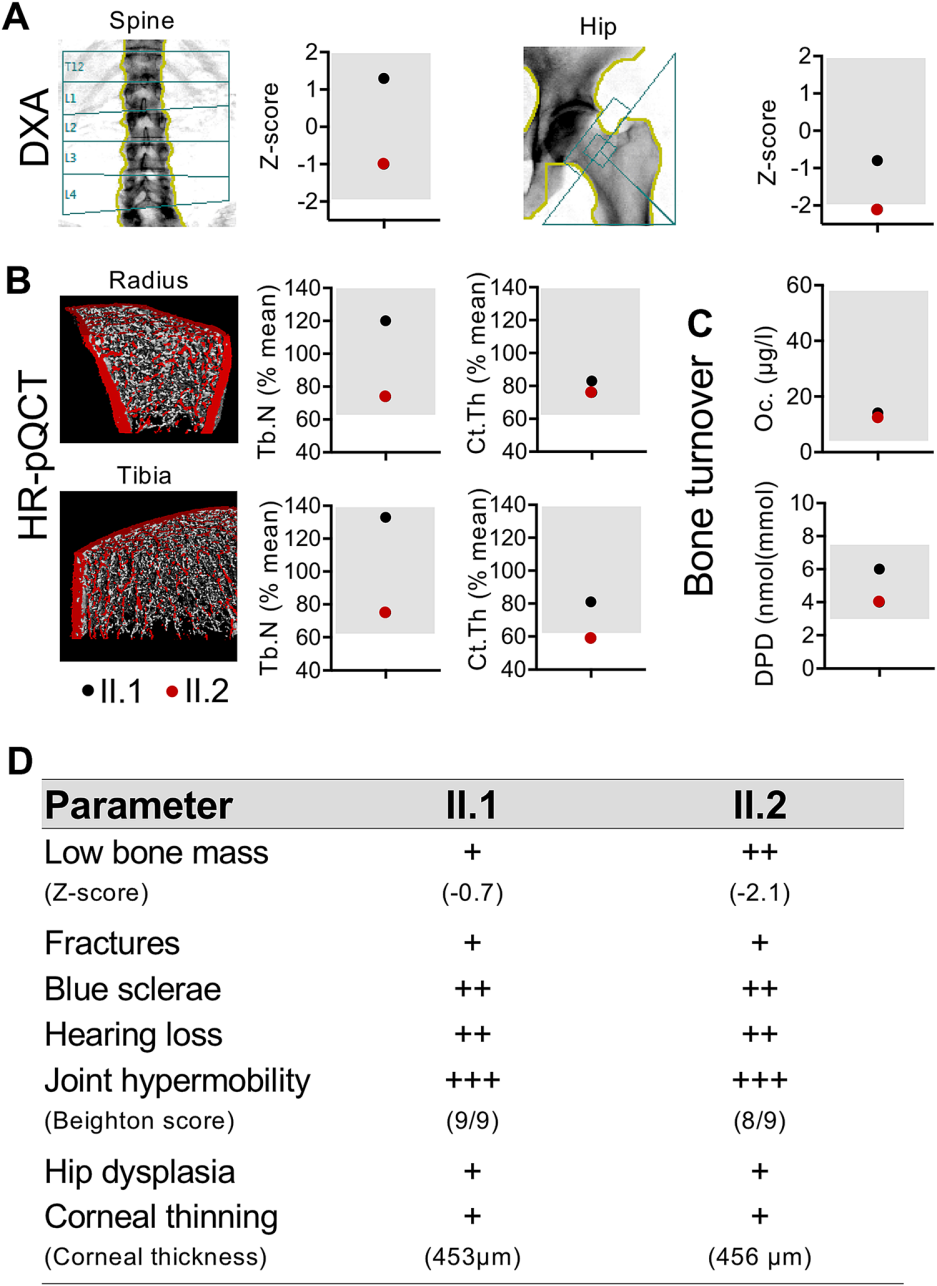
Skeletal analysis. **a**, **b** Bone density and microstructure assessed by DXA at the spine/hip and HR-pQCT at the distal radius/tibia, respectively. HR-pQCT values were compared to sex- and age-matched reference values from the literature [14]. **c** Bone turnover. Oc, osteocalcin, DPD, deoxypyridinoline. Bone-specific alkaline phosphatase values are not shown. Grey boxes indicate reference ranges. Black dot: patient II.1, red dot: patient II.2. **d** Summary of the clinical features in both patients affected by *ZNF469* mutation

DXA and HR-pQCT indicated no clear reductions of bone mass and microstructure compared to previously reported references [14]. In fact, only the BMD Z-score in the hip of patient II.2 was reduced (− 2.1) (Fig. 3a). Next to a slightly reduced trabecular number (Tb.N) in patient II.2 and normal Tb.N in patient II.1 in the distal radius and tibia, we detected a moderately reduced cortical thickness (Ct.Th) in both sisters in the distal radius and tibia (around 60–80% of the age-matched mean [14]) (Fig. 3a, b). Laboratory analyses excluded renal deficiency, hyperparathyroidism or thyroid disorders (Supplementary Table 1). The detected vitamin D deficiency in both sisters was balanced by 20,000 I.E. vitamin D weekly. Markers of bone formation (osteocalcin and BAP) and bone resorption (DPD) were within the normal range for both patients (Supplementary Table 1, Fig. 3c). A summary of the clinical features for both sisters is presented in Fig. 3d.

## Discussion

In this case report, we present two novel compound heterozygous *ZNF469* mutations causing a mild form of brittle cornea syndrome (BCS) that shows clinical resemblance with the extraskeletal manifestations of osteogenesis imperfecta (OI) type I. The phenotype was not only compatible with features of OI but primarily with EDS. In fact, since several other patients with BCS were previously clinically suspected to have a form of EDS [15], BCS was also included in the EDS classification in 2017 [16].

The patients reported here presented with a mild but characteristic phenotype of BCS including blue sclerae, hypermobility, corneal thinning and hearing loss [15]. While previously described as extraskeletal manifestations of OI, blue sclerae or joint hypermobility may also be present in other bone and connective tissue disorders (primarily EDS) [17, 18]. The differential diagnosis of blue sclerae includes particularly connective tissue disorders such as OI, Marfan's syndrome, EDS, and pseudoxanthoma elasticum, among others. The reduction of corneal thickness to around 450 µm in both sisters was moderate but relevant compared to previously published reference values (mean 537.4 µm; 95% confidence interval, 533.8 to 540.9 µm [19]). Nonetheless, patients with severe BCS usually present with a corneal thickness < 400 µm [15]. The auditory phenotype was also mild, however, combined sensorineural and conductive hearing loss was detected in both sisters. Deafness has been reported in two of three unrelated patients with *ZNF469* mutations [20], but there is a wide phenotypic variability with both sensorineural and conductive components [15]. Given the fact that BCS represents a connective tissue disorder, musculoskeletal features have been found in most patients. Especially, hip dysplasia and scoliosis seem to be frequent features of BCS [6, 15]. Hip dysplasia was indeed present in both of our patients, but scoliosis was not diagnosed.

While low bone mineral density has been reported in one family with *ZNF469* mutation [6], we here present the first comprehensive skeletal analysis in patients with *ZNF469* mutations including DXA, HR-pQCT and bone turnover status. Bone mineral density and microstructure were overall not severely reduced, which is reflected by the low fracture rate in the two sisters reported herein. In our patients, the main musculoskeletal feature was hypermobility. In combination with the detected blue sclerae and hearing loss, these characteristics are compatible with EDS as well as the extraskeletal features of OI.

The detected *ZNF469* variants have not been reported before, but similar deletions that lie very close within the *ZNF469* gene have been reported [5, 20]. The majority of known *ZNF469* mutations found in patients with brittle cornea syndrome have a loss-of-function effect [5, 20]. The molecular analysis was of particular importance to make the correct diagnosis and to rule out OI or EDS, especially with regard to the different inheritance pattern (i.e., autosomal dominant in most OI cases). The recurrence risk for BCS in the children of the two probands is negligible due to the recessive inheritance and the extreme rarity of the disorder. Although the variants were ranked as pathogenic, the limitation of this case study includes the fact that no further functional studies were carried out. It is interesting to note that mutations in another member of the ZNF genes, specifically *ZNF687*, have been reported in a peculiar subgroup of severe Paget’s disease of bone associated with giant cell tumor [21, 22].

In conclusion, we demonstrate novel compound heterozygous *ZNF469* mutations in two middle-aged sisters. Our results indicate the existence of a mild phenotype of brittle cornea syndrome with blue sclerae, joint hypermobility and hearing loss, which expands the clinical spectrum of genetic connective tissue disorders that resemble OI and EDS.

## Electronic supplementary material

Below is the link to the electronic supplementary material.

Supplementary file1 (DOCX 14 kb)
