# Supplementary material for: Whole-Exome Sequencing Identifies Novel Compound Heterozygous ZNF469 Mutations in Two Siblings with Mild Brittle Cornea Syndrome
Source: Calcif Tissue Int. 2020 Jul 15;107(3):294–9. doi: 10.1007/s00223-020-00721-3 (PMC7415034; doi:10.1007/s00223-020-00721-3)
Supplement: Supplementary file 1 — Supplementary file1 (DOCX 14 kb) [file 223_2020_721_MOESM1_ESM.docx]

**Supplementary Table 1: Laboratory results in both sisters affected by brittle cornea syndrome (BCS).**

| **Parameter** | **II.1** | **II.2** | **Reference** |
| --- | --- | --- | --- |
| Creatinine (mg/dl) | 0.69 | 0.64 | 0.5-1.0 |
| AP (U/l) | 56 | 55 | 35-104 |
| Ca (mmol/l) | 2.28 | 2.21 | 2.13-2.63 |
| 25-OH-D_3_ (µg/l) | 29.9 (-) | 25.2 (-) | >30 |
| P (mmol/l) | 1.01 | 0.97 | 0.77-1.50 |
| PTH (ng/l) | 53.1 | 66.6 | 17-84 |
| TSH (mU/l) | 3.83 | 2.23 | 0.27-4.20 |
| Osteocalcin (µg/l) | 12.1 | 12.6 | 5.4-59.1 |
| b-ALP (µg/l) | 6.6 | 9.4 | 5.2-24.4 |
| DPD cross links (nmol/mmol) | 6 | 4 | 3-7 |

AP: Alkaline phosphatase, Ca: Calcium, 25-OH-D_3_: 25-Hydroxyvitamin D, P: Phosphate, PTH: Parathyroid hormone, TSH: Thyroid-stimulating hormone, b-ALP: Bone-specific alkaline phosphatase, DPD: Deoxypyridinoline.
